# Influence of patient characteristics on perceived risks and willingness to take a proposed anti-rheumatic drug

**DOI:** 10.1186/1472-6947-13-89

**Published:** 2013-08-12

**Authors:** Richard W Martin, Kelsey McCallops, Andrew J Head, Aaron T Eggebeen, James D Birmingham, Donald J Tellinghuisen

**Affiliations:** 1 Department of Medicine, Rheumatology, Michigan State University, College of Human Medicine Grand Rapids, Grand Rapids, MI 49546, USA; 2 Department of Psychology, Calvin College Grand Rapids, Grand Rapids, MI 49546, USA

**Keywords:** Decision-making, Risk perception, Depression, Health disparity, Disease-modifying anti-rheumatic drugs, Rheumatoid arthritis

## Abstract

**Background:**

The causes of the underutilization of disease modifying anti-rheumatic drugs (DMARDS) for rheumatoid arthritis (RA) are not fully known, but may in part, relate to individual patient factors including risk perception. Our objective was to identify the determinants of risk perception (RP) in RA patients and predictors of their willingness to take a proposed DMARD (DMARD willingness).

**Methods:**

A cross-sectional mail survey of RA patients in a community rheumatology practice. Patients were presented a hypothetical decision scenario where they were asked to consider switching DMARDs. They evaluated how risky the proposed medication was and how likely they would be to take it.

**Results:**

The completed sample included 1009 RA patients. The overall survey response rate was 71%. Patient characteristics: age 61.6 years (range 18-93), 75% female, minority 6.5%, low or marginal health literacy 8.8%, depression 15.0%, duration RA 13.1 years (range 0.5 – 68). Regression models demonstrated that health literacy, independent of low educational achievement or other demographic (including race), was a common predictor of both RP and DMARD willingness. There was partial mediation of the effects of HL on DMARD willingness through RP. Depression and happiness had no significant effect on RP or DMARD willingness. RP was influenced by negative RA disease and treatment experience, while DMARD willingness was affected mainly by perceived disease control.

**Conclusions:**

Risk aversion may be the result of potentially recognizable and correctable cognitive defect. Heightened clinician awareness, formal screening for low health literacy or cognitive impairment in high-risk populations, may identify patients could benefit from additional decision support.

## Background

The prescription of a DMARD for patients with RA is considered a standard of effective care [1] and there is increasing expectation for rheumatologists to objectively measure RA disease activity and to strive to treat patients to achieve low disease activity [2,3]. However a recent study of Medicare managed care enrollees found only 63% received a DMARD [4]. The explanation for underutilization is not fully known; however DMARD decisions are complex and require patients to consider important tradeoffs. DMARDs improve arthritis symptoms and slow the rate of joint damage, but also carry the risk of serious infections amongst other possible harms [5,6].

When patients consider medication information they process the risks affectively as well as cognitively [7]. Differing responses like confidence or anxiety can influence willingness to take a medication and adherence [8]. A good decision is informed, consistent with patient values and acted on [9]. However, this depends on an individual’s ability to understand and evaluate options and to make judgments that are relatively free of bias [10]. There is evidence that a depressed person's diminished ability to think, concentrate or indecisiveness may impair ability to participate in decision-making [11]. Risk assessments can be biased by emotion in part through increased use of the affect heuristic in judging risks rather than more deliberate processing of data [12]. However the specific effects of depression, happiness, and cognition on risk perception have been only incompletely studied in medical decisions. In this study we evaluate how patient demographics, RA disease and treatment related experience, mood and health literacy influence risk perception and DMARD willingness.

The integrated model of behavioral prediction (IMBP) proposes that peoples’ willingness to take a medication are influenced by their beliefs about the expected outcomes of therapy, their social network’s support of and use of the medication (perceived norm), as well their perceived ability to implement it (self-efficacy) [13]. In the model, these three factors plus background variables such as demographics, individual differences, and exposure to health messages (i.e. decision aids) can be used to predict patients’ willingness to take a proposed medication. One would expect if environmental factors do not impede medication use, a patient with positive treatment expectations would have greater willingness to take a proposed medication. We hypothesized that risk perception could be a unique patient attribute that might be influenced by background factors as well as modified by varied formats of a risk presentation in a decision aid.

## Methods

### Design and setting

We conducted a single center, cross-sectional mail survey of a community rheumatology practice. Prior to any study interventions the research protocol was reviewed ruled exempt by the Michigan State University Institutional Review Board. The study was a randomized, single blind, factorial experimental design [14]. The sample frame was created from the practice electronic health record registry and included patients having received care between March 1, 2010 and February 28, 2011 and who were billed under the ICD-9 code 714.0 (RA). 1436 patients were identified in total. All patients were included in the survey. In accordance with the research provision of the practice HIPAA statement, which all patients had received at the time of care, all patient records were reviewed and the most recent Health Assessment Questionnaire 2 score [15] and Clinical Disease Activity Index [16] were extracted and written on the last page of the questionnaire. To maintain complete confidentiality the questionnaire was anonymous. All patients received a 3 contact mail survey using the methods described by Dillman [17].

### Patients

The population was men and women with RA treated in community rheumatology practice.

### Study measurements

The survey instrument assessed the following patient variables:

#### Demographics

Age, gender, ethnicity – race, education, and Medicaid eligibility were recorded. Participants who reported having less than a high-school graduation were classified as having low education. Participants who reported to be of Hispanic, African, or Native American decent were classified as having minority status. Medicaid eligibility was used as an indicator of low-income status.

#### RA and DMARD related experience

RA disease duration, past as well as current DMARD usage and duration of use were elicited. Patient appraisal of current RA control was assessed with the item, “How satisfied are you with the current control of your rheumatoid arthritis?” This was formatted as a 5-point Likert scale anchored with 1 corresponding to “not at all” and 5 as “completely satisfied”. To identify patients who had experienced a past DMARD related serious adverse event, patients were asked, “ Have you ever experienced a side effect from a DMARD that was serious enough that you were hospitalized?” We assessed the level of current DMARD related side effects with the item, “Thinking about the DMARD you most recently started. Are you having any side effects from it that bother you now?” [18]. Finally we evaluated decision regret of their most recent DMARD choice with *the Decision Regret Scale*[19]. This is a 5 item index that evaluated the presence of regret or remorse of a decision made. Post- decision regret has been found to correlate strongly with satisfaction with decision and decisional conflict [19].

#### RA disease status

The *Health Assessment Questionnaire 2* (HAQ 2) [15], a validated derivation of the Stanford modified Health Assessment Questionnaire, which measures functional impairment, was utilized as an indicator of RA severity. The *Clinical Disease Activity Index* (CDAI) is a measure of RA disease activity that sums a physician derived 28 joint swollen and tender joint count, physician global assessment of disease activity and patient global assessment of disease activity into a single, continuous, composite measure of RA disease activity that ranges from 0-76 [16]. A score of 0-10 is classified as low, 11-22 moderate, and ≥23 high disease activity.

#### Happiness

Happiness was evaluated with a single 9 point Likert scale, “Taken all together, how would you say things are these days?” [20].

#### Depression

Subjects completed the *Patient Health Questionnaire-2* which is a two question screening instrument used to evaluate depressive symptoms [21]. In a validation sample population with a prevalence of 7% of major depressive disorder or 18% any depressive disorder the sensitivity and specificity were 82.9% and 90.0% respectively to identify patients with major depressive disorder and 62.3% and 95.4% and respectively to identify patients with any depressive disorder [21].

#### Health literacy

Health literacy (HL) is a measure of multiple domains including: reading fluency, ability to locate and use information and do simple mathematical tasks. We conceptualized HL as not only a measure useful for classifying patient’s reading fluency, but also as a broader descriptive indicator of cognitive function including recall and critical thinking [22]. We used a validated three question screening index to identify subjects with inadequate or marginal HL [23]. A score of ≥ 9 on difficulty using health information has been reported to differentiate patients with low or marginal HL as measured by the gold standard Test of Functional Health Literacy in Adults [24]. To aid readers interpret the findings, we transposed the health literacy score, such that lower score reflects lower health literacy.

#### Risk perception and willingness to take a proposed medication

We presented patients a hypothetical decision scenario where they were asked to consider switching DMARDs. Patients were randomized to read 1 of 4 variations of the format of risk and safety information, which were derived from an existing DMARD patient decision aid [25]. The hypothetical scenarios differed in two ways: SIE risk level and simultaneous presentation or omission of safety information with the risk statement. We wanted to evaluate patient perception over a range of risk levels from what would be considered a relatively low risk of having a DMARD related SIE to a relatively high risk which was above the societally determined acceptable levels. Equal numbers were presented a decision scenario where the proposed DMARD had a risk of developing a SIE of 1% or 8%. Similarly, equal numbers were randomized to be presented safety-monitoring procedures to reduce risk of SIE simultaneously (yes or no). In the regression analyses risk and context were defined as control variables. An example of the low risk with contextual safety information condition is depicted in the online supplement. Patients who had a likelihood to take the proposed medication rating greater than 50% (≥ 5 on a 1-9 scale) were classified as an “intender” and those with a rating of ≤ 4 were classified as a “non-intender” [13].

### Analysis

Descriptive analyses were performed to provide information on the characteristics of the respondents. Bivariate relationships were evaluated with Pearson correlation coefficients and independent samples t-tests as appropriate. Differences in categorical variables were evaluated in 2×2 tables calculating odds ratios. We created linear regression models from the data to identify patient characteristics that predicted RP and DMARD willingness. Finally we evaluated the causal hypothesis that RP mediates the effects of HL on DMARD willingness using methods described by Baron and Kenny [26,27]. All analysis was performed with IBM SPSS Statistics version 19.0 [28].

## Results

1436 patients with RA were surveyed. All participants were randomized and allocated to intervention. Subjects were blinded to allocation. Data were analyzed on 1009 respondents. In addition to non-respondents, we learned that 4 patients had died, 4 had changed address and could not be reached, and 5 returned the questionnaire without completion. The overall response rate was 71%. Data from all study completers were included in the analysis. In the completed sample all patients had RA. For RP and DMARD willingness, missing data were 11.2% and 10.0% respectively. To evaluate if participants with missing values were systematically different than cases without missing values [29], we evaluated means of missing values by estimation-maximization methods. Little’s MCAR test Chi-Square was P <.01 suggesting that missing values are not missing completely at random [30]. Review of estimated means of RP and DMARD willingness disclosed as expected that there were higher rates of missing values in patients with low health literacy (11.2 vs. 21.8% and 10.0 vs. 18.4% respectively), low education (11.2 vs. 17.7% and 10.0 vs. 14.5% respectively) and major depression (11.2 vs. 15.4% and 10.0 vs. 12.6% respectively), but not in female, low income or minority subjects. As missing values were not missing completely at random, multiple imputation was utilized. SPSS performed imputation for variables with > 10.0% missing values [30].

### Univariate analysis

Descriptive analysis was performed to provide information about the general characteristics of the study populations and is presented in Table 1.

**Table 1:** Patient characteristics

**Demographics**			
	**Mean**	**S.D.**	**Range**
Age (years)	61.52	13.37	18-93
Female sex	73.2%		
Minority	6.5%		
Medicaid	18.6%		
Less than high school graduation	12.3%		
**Disease and Treatment Related Experience**			
RA duration (years)	13.15	11.18	1-68
Number of DMARDs taken	2.45	1.54	0-11
Past or current biologic DMARD use	53.2%		
TNF related knowledge	8.39	2.45	1-14
Past hospitalization with SAE	4.0%		
Current bother from DMARD side effects	1.52	0.91	0-5
Satisfaction with RA control	3.94	0.97	1-5
Support to take DMARD	13.31	0.50	3-15
Decision regret of current DMARD choice	8.11	23.38	5-25
**RA Disease Status**			
HAQ 2 disability	0.76	0.64	0-3
Clinical disease activity index	11.82	8.41	0-54
**Mood and Cognition**			
Major depression	15.0%		
Happiness	6.86	1.77	0-9
Health literacy	13.32	2.51	3-15
Low or marginal health literacy	8.8%		

### Bivariate analysis

Correlations of continuous patient characteristics with RP and DMARD willingness were computed. Risk Perception was significantly (P < 0.05) positively correlated with age, HAQ disability, CDAI, bother from current DMARD side effects, depressive symptoms and post-decision regret RP was significantly (P < 0.05) negatively correlated with health literacy, TNF knowledge, satisfaction with current control of RA, happiness, and risk perception. Willingness to take a proposed medication was significantly positively correlated with health literacy, number of past DMARDS taken, TNF knowledge, and satisfaction with current control of RA, DMARD willingness was. There were significantly negatively correlated with age and post-decision regret with DMARD willingness. Independent sample t-tests disclosed significant (P<0.01) between group differences in RP in patients who were depressed, had past experience of a DMARD related serious adverse event, or were classified as low income or low educational status. There were no significant between group differences in RP by recent onset RA (≤ 3 years), gender, minority status, or past biologic use.

We explored differences between intenders and non-intenders. Overall 31.2% of patients were classified as non-intenders. They were significantly older, had experience using fewer DMARDs in the past, had lower health literacy, less knowledge about TNF inhibiting DMARDs, had higher levels of post-decision regret related to the DMARD they had most recently initiated, as well as and higher perception of medication risk. There was no difference in happiness, depressive symptoms, HAQ disability or CDAI. When compared to normal HL patients, low or marginal HL respondents were significantly more likely to be non-intenders with an odds ratio (OR) of 2.39 (95% C.I.1.27 -3.90). Patients with major depression were no more likely to be non-intenders compared to non-depressed patients with an OR of 1.17 (95% C.I. 0.79-1.76). Minority and low-income patients were no more likely to be non-intenders compared to non-minority or low-income patients with an OR of 1.22 (95% C.I. 0.70-2.15) and OR of 0.76 (95% C.I. 0.54 -1.08) respectively.

### Multivariate analysis

The integrative model of behavioral prediction (IMBP), proposes that a limited number of variables can be identified to explain a substantial portion of the variance of a behavior. In the IMBP theorizes that willingness to perform a target behavior follows from specific beliefs (rational or irrational) and attitudes a person has about that behavior [13]. In our survey willingness to take a proposed DMARD is the target behavior.

We developed linear regression models to identify predictors of RP and DMARD willingness from a broad pool of variables. These included the survey version control variables, as well as demographics, RA disease status, RA and treatment related experience, as well as mood and HL.

Risk Perception and Patient Characteristics: Results of hierarchical linear regression modeling of predictors of risk perception are presented in Tables 2 and 3. The overall model explained 13.5% of the variation of risk perception which is considered a moderate effect size [31]. The standardized regression coefficients show after controlling for risk level, the strongest predictor of RP was HAQ2 disability, followed by HL, and current or past experience of DMARD related AE. Age, TNF knowledge, happiness and depression, and other demographics did not significantly add to the predictive power of the model.

**Table 2:** Hierarchical regression model summary‒predictors of risk perception

						**Change statistics**	
**Model**	**Predictors**	**R**	**R**^**2**^	**Adjusted R**^**2**^	**Std. error of the estimate**	**R**^**2 **^**change**	**Sig. F change**
1	Survey version control variables	.138	.019	.013	2.1759	.019	0.05
2	Demographics	.176	.031	.010	2.1794	.012	NS
3	RA disease status	.228	.052	.025	2.1626	.021	NS
4	Disease & treatment experience	.331	.109	.057	2.1270	.057	0.02
5	Mood	.334	.111	.053	2.1315	.002	NS
6	Health Literacy	.367	.135	.075	2.1066	.023	0.021

**Table 3:** Regression coefficients of predictors of risk perception

**Predictor**	**Unstandardized coefficients**		**Standardized coefficients**	**t**	**Sig**
	**B**	**Std. error**	**Beta**		
(Constant)	-.438	2.290		-.191	NS
Survey version ‒safety context	.110	.243	.025	.454	NS
Survey version‒risk level	.708	.247	.161	2.870	.004
HAQ2 Disability	.517	.230	.152	2.252	.025
Current bother from AE due to most recently initiated DMARD	.353	.146	.146	2.418	.016
Past DMARD related SAE	1.303	.599	.129	2.175	.030
Health literacy	-.134	.057	-149	2.368	.018
Satisfaction with control of RA	.136	.190	.055	.718	NS
Post-Decision Regret	.043	.045	.065	.957	NS
Depression	-.112	.391	-.018	-.286	NS
Happiness	.006	.100	.005	.064	NS

N.S. = Age, female sex, minority, low income, low education, CDAI, duration RA, number of past DMARDs used, past or present biologic use, and TNF knowledge.

DMARD Willingness and Patient Characteristics: Results of hierarchical linear regression modeling of predictors of DMARD willingness are presented in Tables 4 and 5. The overall model explained 12.7% of the variation of likelihood to take the proposed medication which is considered a moderate effect size [31]. The collinearity statistics were within acceptable ranges with all tolerance >0.4 and Variance Inflations factors < 2.2. The standardized regression coefficients show the strongest predictors of DMARD willingness were satisfaction with control of RA and regret related to their previous DMARD choice, followed by HL, and the level and context of the risk being presented. Age and other demographic characteristics, extent of past RA and general DMARD related experience, happiness and depression did not significantly add to the predictive power of the model.

**Table 4:** Hierarchical regression model summary‒predictors of willingness to take proposed DMARD

						**Change statistics**	
**Model**	**Predictors**	**R**	**R**^**2**^	**Adjusted R**^**2**^	**Std. error of the estimate**	**R**^**2 **^**change**	**Sig. F change**
1	Survey version control variables	.188	.035	.029	2.5080	.035	.01
2	Demographics	.215	.046	.025	2.5135	.011	NS
3	RA disease status	.220	.049	.021	2.5183	.002	NS
4	Disease & treatment experience	.331	.100	.057	2.4718	.061	0.2
5	Mood	.336	.113	.055	2.4752	.003	NS
6	Health Literacy	.356	.127	.066	2.4599	.014	.03

**Table 5:** Regression coefficients of predictors of willingness to take proposed DMARD

**Predictor**	**Unstandardized coefficients**		**Standardized coefficients**	**t**	**Sig**
	**B**	**Std. error**	**Beta**		
(Constant)	8.810	2.675		3.294	.001
Survey version-safety context	-.654	.283	-.129	2.306	.022
Survey version-risk level	-.749	.288	-.147	2.599	.010
Satisfaction with control of RA	-.483	.222	-.167	2.175	.030
Post-Decision Regret	-.125	.053	-.160	2.356	.019
Depression	-.305	.457	-.041	.668	NS
Happiness	-.020	.117	-.014	.170	NS
Health literacy	.154	.071	.149	2.186	.030

N.S. = Age, female sex, minority, low income, or low education status, HAQ2 Disability, CDAI, duration RA, number of past DMARDs, past or current biologic use, TNF knowledge, past DMARD related SAE, current Bother From AE d/t most recently initiated DMARD, and DMARD support.

### Mediation analysis

**Figure 1:**
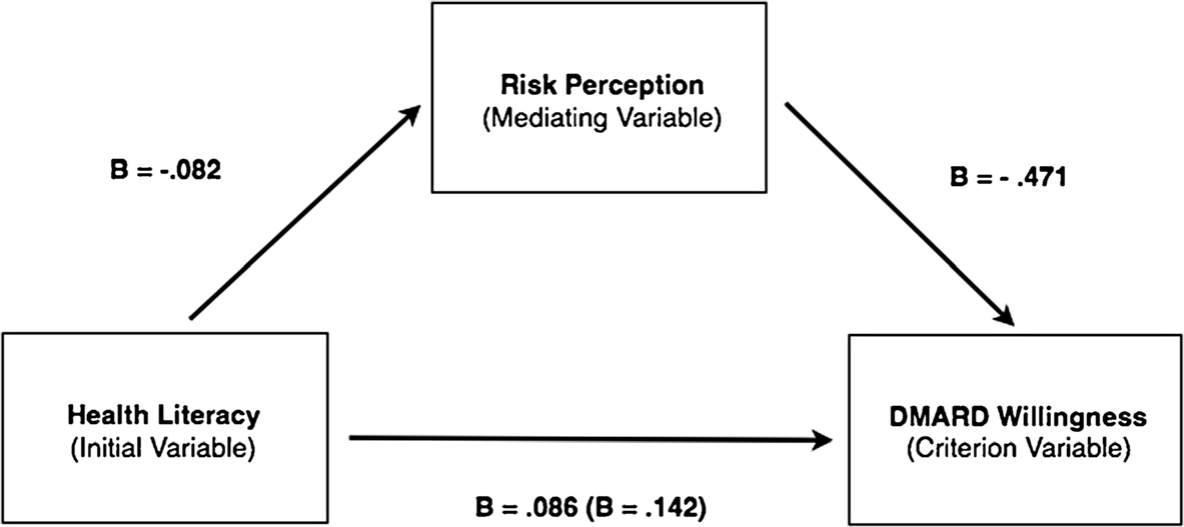
Mediation model.

In regression analysis reduced HL was significantly associated with increased risk perception and decreased DMARD willingness. Though a cross sectional survey and correlational research methods are not optimal approaches to evaluate causation, we wanted to evaluate the degree to which HL’s effect on DMARD willingness was mediated through risk perception. We conceptualized this with a simplified causal model illustrated in Figure 1.

First we evaluated the relationship between HL and DMARD willingness. This demonstrated there was a significant effect to be mediated, β = .142 (P< 0.01). Next we evaluated the effect of the initial variable, HL, with the mediator, RP, which displayed significant relationship, β = - .082 (P<0.01). Finally we demonstrated the mediating effect of RP on DMARD willingness, the criterion variable, by simultaneously using RP and HL to predict DMARD willingness. This revealed a significant relationship between RP and DMARD willingness, β = - .471 (P<0.01). Once this mediator was taken into account, the relationship between HL and DMARD willingness dropped to β = .086 (P<.01). This indicates that RP partially mediates the relationship between HL and DMARD willingness.

## Discussion

Based on examination of variation of practice patterns in the Dartmouth Atlas, John Wennberg, M.D. has suggested three general approaches to reduce inappropriate variation and increase the equity of medical care: increase the amount of effective care, reduce supply sensitive care and increase preference sensitive care [32]. The underuse of DMARDs by patients with RA, is an example of underutilization of effective care. This may be explained by healthcare system failures, individual physician behavior, or in some cases subsets of patients who are informed but elect not to take medications recommended in national standards of care. A recent computer based, simulated decision, discrete choice experiment of 144 RA patients’ preferences of specific risks and benefits of treatment found that when compared to white adults with RA, African American RA patients were more likely to be classified as risk averse (OR 8.4 [3.1-23.1]) [33]. In general, it is unknown if risk aversion is an inherent culturally based trait, influenced by affect, the result of modifiable deficits in knowledge or arises from potentially correctable cognitive bias [34]. The purpose of our study was to identify, in a large community cohort of RA patients, if a limited number of patient characteristics could predict patient risk perception and how this influenced the likelihood to take a proposed DMARD.

In our analysis, guided by the Integrative Model of Behavioral Prediction [13], we developed regression models to identify the determinants of risk perception and predictors of patent's willingness to take the proposed medication (DMARD willingness). In these we considered background variables (demographics, socio-economic, disease and DMARD experience, disease activity and related disability, support to acquire and administer DMARDS) and patient beliefs about outcomes of their current DMARD use (adverse events and satisfaction with their disease control). The results create a valid model, which accounts for significant proportion of the variance of risk perception and likelihood to take a proposed medication. RP and DMARD willingness are highly correlated, but distinct attributes, which are predicted by health literacy as well as different aspects of patients’ DMARD and disease experience. We found that when risk level and context were controlled for, RP was influenced by patient functional impairment / RA severity, past experience of a DMARD related serious adverse event, current bother from DMARD related side effects, as well as HL (see Table 3). In contrast DMARD willingness, when controlled for risk level and context, was predicted by satisfaction with RA control, post-decision regret related to the most recent DMARD choice, and HL (see Table 5). HL was the only thread that contributed to both predictive models. Importantly, depression and happiness did not demonstrate a significant effect on either criterion variable. It is notable age, gender, minority status, low education, low-income status and TNF knowledge did not contribute to the prediction of DMARD willingness. Although our data creates a significant model, it is important to note that it explains only 12.7% of the variance of willingness to take a proposed medication. This suggests that other unmeasured variables i.e. doctor patient relationship, more nuanced measurement of age related cognitive impairment, perceived costs, individual values of benefit compared to risk could significantly add to the predictive power of the model.

The results do demonstrate that HL, independent of low educational achievement or other demographics, is a common predictor of both RP and DMARD willingness. Our meditational analysis suggests that much of the effect of HL on DMARD willingness was mediated through RP. The clinical implication is that HL, as an indicator of cognition, may provide an identifiable predictor of risk aversion that could be accommodated through decision support to minimize cognitive bias [35-37]. In our study population, the 8.8% prevalence of low or marginal HL, as measured by a simple 3 question survey, mirrors Shin and colleagues who found utilizing the ACR Neuropsychological Battery the prevalence of cognitive impairment in RA patients ranged from 8% (semantic fluency test) to 29% (visuo-spatial learning/memory test) [38]. This highlights the frequency with which rheumatology providers encounter such high-risk patients, who may not be identified by demographic characteristics.

When making treatment decisions, patients may use one or more cognitive strategies. Prospect theory suggests that decision makers carefully weigh multiple attributes of available treatment options before arriving a rational choice that maximizes benefit [39]. This is theorized to rely on “System 2” which is the mode of thinking that directs conscious, deliberate, effortful activities like choice [40]. Alternatively patients may utilize a heuristic approach, making simpler more cognitively efficient decisions using rules of thumb. This is thought to be directed by “System 1” which is the mode of thinking that is unconscious, fast, intuitive and the source of impressions and feelings at the source of many of our explicit beliefs [40]. In the setting of patients evaluating a new DMARD, this might be conceptualized as a tension between risk as feelings versus risk as analysis [41]. When a patient with reduced HL engages in a conversation with a physician evaluating medication options, the constrained time increases cognitive effort. Cognitive overload could influence their evaluation of risk and willingness to take the proposed DMARD leading them to substitute rational deliberation of the facts with the use of a easier, faster heuristics based on their past RA and DMARD experiences [40]. Examples might include: “if I had a side effect before it will happen again” or accepting the default “I’ll do what the doctor suggests”. This proposal is consistent with the findings that our patients’ risk perception was heavily influenced by experience of greater functional disability as well as past and present DMARD side effects. In a previous study we also found that patients’ trust in physician had nearly seven times the effect on their confidence in a DMARD decision than any other predictor including numeric literacy and DMARD knowledge [18]. This offers further evidence that many patients rely on non-deliberative decision strategies when choosing a DMARD.

The findings should be interpreted given the limitations of the study. Most notable is that our survey provided a simulated decision rather than observing an actual DMARD choice prospectively in the clinic. However, vignettes have been used in many previous studies to validly simulate patient and physician decisions [14,42]. In addition if a prospective study design had been utilized, time and cost would have made it impossible to accrue 1009 patient responses, which provided the statistical power to evaluate multiple predictor variables each with small effects. The selection of background and predictor variables was guided by a widely accepted psychological/health behavior theory [13] and included an objective physician collected RA activity measure, the CDAI, plus patient reported outcomes of disability, appraisal of disease control as well as DMARD side effects, TNF knowledge, and validated screening measures for depression and HL. To differentiate patients with varied perception of medication risk, we utilized a simulated decision scenario with high and low risk as well as two different safety monitoring framing contexts. The response rate was high at 71%, however as the survey was anonymous, we were not able to compare the characteristics of responders to non-responders, and it remains possible that there was a differential non-response in subgroups of patients. Still the findings are important as with 6.5% minority and 18.6 % Medicaid respondents, our completed sample was demographically similar to a previous large random sample of RA patients receiving care in five geographically dispersed community rheumatology practices in Michigan [18], thus the results are likely generalizable to similar populations. Finally a cross-sectional survey allows the description of the association of criterion and predictor variables but is not the strongest design to assert causation between variables.

## Conclusion

In our large community-based sample, medication risk perception seemed to be driven by negative RA disease and treatment experience, while DMARD willingness was predicted by perceived disease control. In the comparison of intenders and non-intenders, non-intenders were significantly older, had experience using fewer DMARDs, had lower health literacy, less knowledge about TNF inhibiting DMARDs, had higher levels of post-decision regret related to the DMARD they had most recently initiated, as well as higher perception of medication risk. Minority, low-income, and depressed patients were no more likely to be non-intenders. While clinicians should be alert to mood disturbance, depression may have less effect on risk perception and decision-making than previously proposed [10-12]. While racial disparities with the under-utilization of DMARDs exist in the US, our data support a premise that risk aversion could be, in many cases, the result of a potentially recognizable and correctable cognitive defect or bias rather than an inherent, cultural trait.

When proposing to initiate a new DMARD, clinicians must be alert for the possibility of cognitive impairment whether derived from educational, language, age related decline in information processing or sensory defect that could effect the patient’s decision making. Time pressures increase cognitive load, which might justify extending the time of deliberation beyond the constraints of the office visit by scheduling a follow up visit, using decision supports like decision aids, involving family members, or providing post-visit coaching by other health professionals [43]. Heightened clinician awareness, formal screening for low health literacy or cognitive impairment in high-risk populations, may identify patients could benefit from additional decision support. Further investigation is needed to evaluate if the use of patient decision aids to extend and structure deliberation, can increase the use effective care by the adoption of recommended medications.

## Competing interests

The authors’ declare that there are no competing interests.

## Authors’ contributions

All authors in this study were involved in conception and design, critical revision of the manuscript for important intellectual content, and gave final approval of the manuscript for submission. RWM, AJH, ATE, JDB participated in data collection. RWM, KM, DT performed the data analysis. All authors read and approved the final manuscript.

## Pre-publication history

The pre-publication history for this paper can be accessed here:

http://www.biomedcentral.com/1472-6947/13/89/prepub
